# Crystal structure of penoxsulam

**DOI:** 10.1107/S2056989017011458

**Published:** 2017-08-08

**Authors:** Hyunjin Park, Jineun Kim, Hojae Chiang, Tae Ho Kim

**Affiliations:** a Department of Chemistry (BK21 plus) and Research Institute of Natural Sciences, Gyeongsang National University, Jinju 52828, Republic of Korea

**Keywords:** crystal structure, sulfonamide herbicides, triazolo­pyrimidine herbicides, penoxsulam

## Abstract

The title compound, C_16_H_14_F_5_N_5_O_5_S [systematic name: 2-(2,2-di­fluoro­eth­oxy)-*N*-(5,8-dimeth­oxy-1,2,4-triazolo[1,5-*c*]pyrimidin-2-yl)-6-(tri­fluoro­meth­yl)benzene­sulfonamide], is used as a herbicide. The asymmetric unit of this structure comprises two independent mol­ecules, *A* and *B*. The dihedral angles between the ring planes of the triazolo­pyrimidine ring systems and the benzene rings are 68.84 (7)° for *A* and 68.05 (6)° for *B*. In the crystal, weak inter­molecular π–π inter­actions, with centroid–centroid separations of 3.4456 (17) and 3.5289 (15) Å and C—F⋯π [3.5335 (17) Å and 107.92 (13)°] contacts link adjacent mol­ecules into chains along [001]. C—H⋯O and C—H⋯F hydrogen bonds link type *B* mol­ecules into chains parallel to (100). Additional C—H⋯F hydrogen bonds together with short F⋯F contacts further aggregate the structure into a three-dimensional network.

## Abstract 2

The herbicide penoxsulam crystallizes with two independent mol­ecules in the asymmetric unit. The crystal structure is stabilized by C—F⋯π and π–π inter­actions that combine with C—H⋯F and C—H O hydrogen bonds and weak inter­molecular F⋯F short contacts to form a three-dimensional network.

## Chemical context   

Penxosulam is a triazolo­pyrimidine sulfonamide herbicide, which is used to control the growth of annual grasses, sedges, and broadleaf weeds in rice agriculture. The compound inhibits the synthesis of acetolactate and targets the biosynthesis of branch-chained amino acids, a metabolic pathway found in plants, fungi, and microorganisms. Acetolactate synthase (ALS) inhibitors are present in most effective herbicides. They are used in agriculture because they show a broad weed control spectrum, crop selectivity, are safe to humans, and can be applied at relatively low usage rates (Jabusch *et al.*, 2005 ▸; Yasuor *et al.*, 2009 ▸). Moreover, penoxsulam controls a number of troublesome weeds including northern jointvetch, alligatorweed, Texasweed/Mexicanweed, annual sedge, ducksalad, smartweed, and hemp sesbania (Willingham *et al.*, 2008 ▸). We now report here the crystal structure of penoxsulam, 2-(2,2-di­fluoro­eth­oxy)-*N*-(5,8-dimeth­oxy[1,2,4]triazolo[1,5-*c*]-pyrim­idin-2-yl)-6-(tri­fluoro­meth­yl)benzene­sulfonamide.
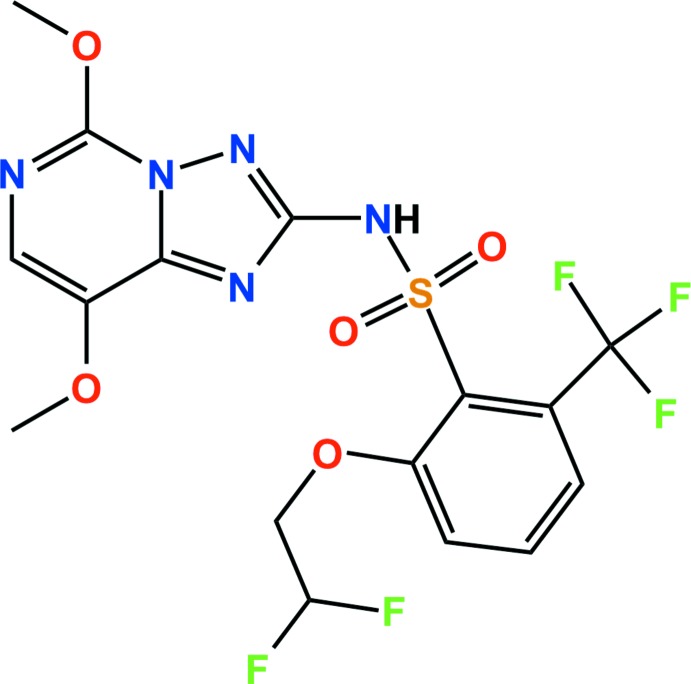


## Structural commentary   

**Figure 1:**
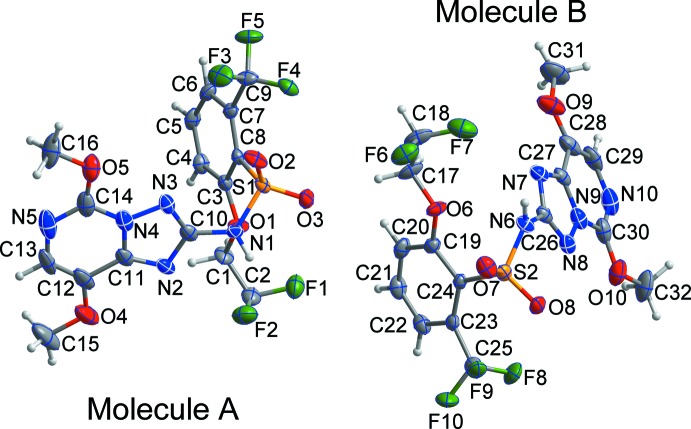
The mol­ecular structures of the title compound with the atom labelling and displacement ellipsoids are drawn at the 50% probability level. H atoms are shown as small spheres of arbitrary radius.

The penoxsulam mol­ecule crystallizes with two independent mol­ecules, *A* and *B* in the asymmetric unit, Fig. 1 ▸. The triazolopyrimidin unit caries meth­oxy substituents while the benzene ring of the benzene­sulfonamide segment of the mol­ecule carries tri­fluoro methyl and the unusual di­fluoro­eth­oxy substituents. The dihedral angles between the planes of the triazolo­pyrimidine ring systems and the benzene ring planes are 68.84 (7)° in mol­ecule *A* and 68.05 (6)° in *B*. All bond lengths and bond angles are normal and comparable to those observed in similar crystal structures for triazolo­pyrimidine (AboulWafa *et al.*, 2014 ▸) and triazolo­pyrimidine sulfonamide herbicides (Kumar *et al.*, 2012 ▸).

## Supra­molecular features   

**Figure 2:**
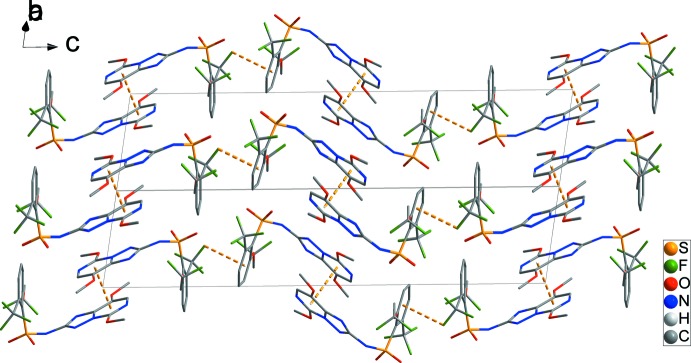
C—F⋯π and weak π–π inter­actions, yellow dashed lines, form chain along [001] in the crystal packing. H atoms have been omitted for clarity.

**Figure 3:**
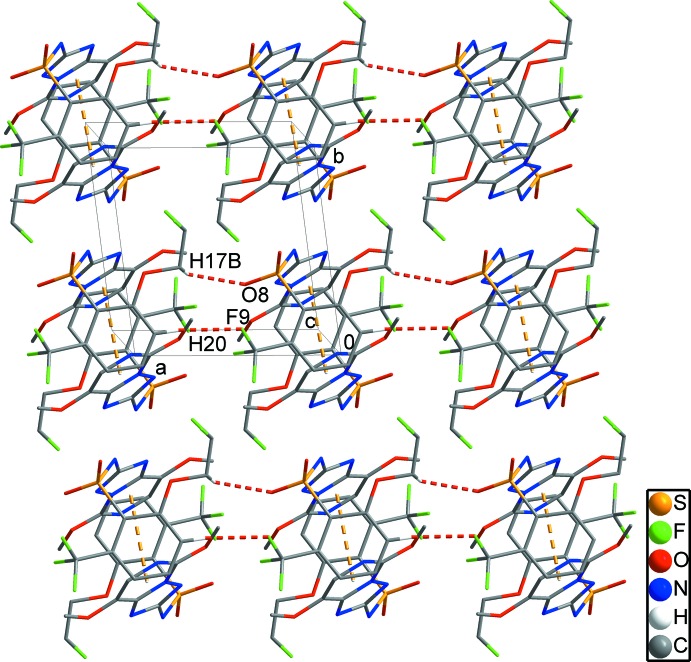
A two-dimensional network in the *ab* plane. Yellow dashed lines indicate weak inter­molecular π–π inter­actions. Red dashed lines indicate C—F⋯π inter­actions and C—H⋯O hydrogen bonds. H atoms have been omitted for clarity.

**Figure 4:**
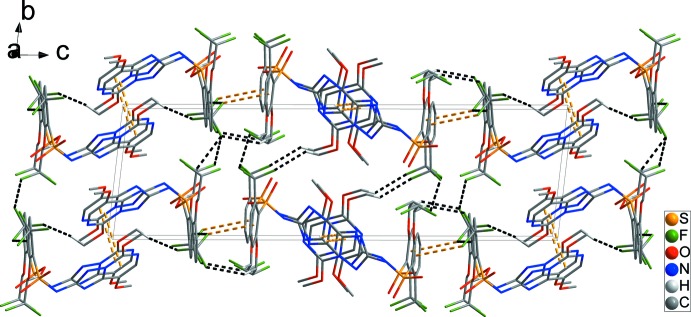
Overall packing showing the three-dimensional network viewed along the *a*-axis direction, C—H⋯F hydrogen bonds and F⋯F inter­molecular short contacts are shown as black dashed lines. H atoms have been omitted for clarity.

In the crystal, there are weak π–π inter­actions between the pyrimidine rings of neighbouring mol­ecules of type *A* with *Cg*1⋯*Cg*1^v^ = 3.4456 (17), and type *B* with *Cg2*⋯*Cg2* ^vi^ = 3.5289 (15) Å [*Cg*1 and *Cg*2 are the centroids of the N4/N5/C11–C14 and N9/N10/C28–C30 rings, respectively; symmetry codes: (v) −*x*, −*y*, −*z* + 1; (vi) −*x* + 2, −*y*, −*z*]. These combine with C25—F9⋯*Cg*3^vii^ inter­actions involving the C3–C8 benzene ring to form chains along [001] (Fig. 2 ▸). C17—H17*B*⋯O8^i^ and C20—H20⋯F9^i^ hydrogen bonds form chains along the *a*-axis direction, forming a two-dimensional network in the *ab* plane (Fig. 3 ▸ and Table 1 ▸). In addition, short inter­molecular F1⋯F5^iii^ and F6⋯F10^iii^ contacts [2.846 (2) and 2.794 (2) Å] together with C1—H1*A*⋯F10^i^, C16—H16*C*⋯F3^ii^, C18—H18⋯F10^iii^ and C32—H32*C*⋯F8^iv^ hydrogen bonds generate a three-dimensional network with mol­ecules stacked along the *a-*axis direction (Fig. 4 ▸) and Table 1 ▸).

## Database survey   

Crystal structures of sulfonamide (Kang *et al.*, 2015 ▸; Chen, Wu *et al.*, 2005 ▸) and triazolo­pyrimidine (Chen, Li *et al.*, 2005 ▸) herbicides have been reported previously. Moreover, the crystal structures of compounds with a triazolo­pyrimidine ring system and a benzene ring in the mol­ecule, ethyl 2-(5,7-dimethyl-1,2,4-triazolo[1,5-*a*]-pyrimidin-2-yl­oxy)benzoate (Chen, Li *et al.*, 2005 ▸) and 5-(4-Chloro­phen­oxy)-6-isopropyl-3-phenyl-3*H*-1,2,3-triazolo[4,5-*d*]-pyrimidin-7(6*H*)-one (Zeng *et al.*, 2009 ▸) have also been reported.

## Synthesis and crystallization   

The title compound was purchased from the Dr. Ehrenstorfer GmbH Company. Single crystals were obtained by slow evaporation of an aceto­nitrile solution.

## Refinement   

Crystal data, data collection and structure refinement details are summarized in Table 2 ▸. All H atoms were positioned geometrically and refined using a riding model with *d*(N—H) = 0.88 Å, *U*_iso_(H) = 1.2*U*_eq_(C) for the N—H group, *d*(C—H) = 0.95 Å, *U*_iso_(H) = 1.2*U*_eq_(C) for aromatic C—H, *d*(C—H) = 0.98 Å, *U*_iso_(H) = 1.5*U*_eq_(C) for methyl group, *d*(C—H) = 0.99 Å, *U*_iso_(H) = 1.2*U*_eq_(C) for C*sp* ^3^—H, and *d*(C—H) = 1.00 Å, *U*_iso_(H) = 1.5*U*_eq_(C) for C*sp* ^3^—H.

## Supplementary Material

Crystal structure: contains datablock(s) I, New_Global_Publ_Block. DOI: 10.1107/S2056989017011458/sj5532sup1.cif

Structure factors: contains datablock(s) I. DOI: 10.1107/S2056989017011458/sj5532Isup2.hkl

Supporting information file. DOI: 10.1107/S2056989017011458/sj5532Isup3.cml

CCDC reference: 1566461

Additional supporting information:  crystallographic information; 3D view; checkCIF report

## Figures and Tables

**Table 1:** Hydrogen-bond geometry (Å, °)

*D*—H⋯*A*	*D*—H	H⋯*A*	*D*⋯*A*	*D*—H⋯*A*
C1—H1*A*⋯F10^i^	0.99	2.48	3.293 (3)	139
C16—H16*C*⋯F3^ii^	0.98	2.40	3.185 (3)	136
C17—H17*B*⋯O8^i^	0.99	2.40	3.102 (3)	127
C18—H18⋯F10^iii^	1.00	2.60	3.104 (3)	111
C20—H20⋯F9^i^	0.95	2.55	3.466 (3)	162
C32—H32*C*⋯F8^iv^	0.98	2.36	3.146 (3)	137

Symmetry codes: (i) 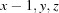; (ii) 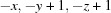; (iii) 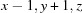; (iv) 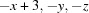.

**Table 2:** Experimental details

Crystal data	
Chemical formula	C_16_H_14_F_5_N_5_O_5_S
*M*_r_	483.38
Crystal system, space group	Triclinic, *P* 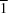
Temperature (K)	173
*a*, *b*, *c* (Å)	8.1945 (3), 8.3733 (3), 28.3277 (9)
α, β, γ (°)	82.698 (2), 84.183 (2), 81.814 (2)
*V* (Å^3^)	1901.43 (12)
*Z*	4
Radiation type	Mo *K*α
μ (mm^−1^)	0.26
Crystal size (mm)	0.40 × 0.19 × 0.11

Data collection	
Diffractometer	Bruker APEXII CCD
Absorption correction	Multi-scan (*SADABS*; Bruker, 2014 ▸)
*T*_min_, *T*_max_	0.646, 0.746
No. of measured, independent and observed [*I* > 2σ(*I*)] reflections	24336, 6571, 5503
*R*_int_	0.032
(sin θ/λ)_max_ (Å^−1^)	0.595

Refinement	
*R*[*F* ^2^ > 2σ(*F* ^2^)], *wR*(*F* ^2^), *S*	0.044, 0.106, 1.05
No. of reflections	6571
No. of parameters	581
No. of restraints	1
H-atom treatment	H-atom parameters constrained
Δρ_max_, Δρ_min_ (e Å^−3^)	0.47, −0.52

Computer programs: *APEX2* and *SAINT* (Bruker, 2014 ▸), *SHELXS97* and *SHELXTL* (Sheldrick, 2008 ▸), *SHELXL2014* (Sheldrick, 2015 ▸), *DIAMOND* (Brandenburg, 2010 ▸) and *publCIF* (Westrip, 2010 ▸).

## supplementary crystallographic information

### Crystal data

**Table:**

C_16_H_14_F_5_N_5_O_5_S	*Z* = 4
*M**_r_* = 483.38	*F*(000) = 984
Triclinic, *P*1	*D*_x_ = 1.689 Mg m^−^^3^
*a* = 8.1945 (3) Å	Mo *K*α radiation, λ = 0.71073 Å
*b* = 8.3733 (3) Å	Cell parameters from 9990 reflections
*c* = 28.3277 (9) Å	θ = 2.5–27.3°
α = 82.698 (2)°	µ = 0.26 mm^−^^1^
β = 84.183 (2)°	*T* = 173 K
γ = 81.814 (2)°	Plate, colourless
*V* = 1901.43 (12) Å^3^	0.40 × 0.19 × 0.11 mm

### Data collection

**Table:**

Bruker APEXII CCD diffractometer	5503 reflections with *I* > 2σ(*I*)
φ and ω scans	*R*_int_ = 0.032
Absorption correction: multi-scan (SADABS; Bruker, 2014)	θ_max_ = 25.0°, θ_min_ = 1.5°
*T*_min_ = 0.646, *T*_max_ = 0.746	*h* = −9→9
24336 measured reflections	*k* = −9→9
6571 independent reflections	*l* = −32→33

### Refinement

**Table:**

Refinement on *F*^2^	1 restraint
Least-squares matrix: full	Hydrogen site location: inferred from neighbouring sites
*R*[*F*^2^ > 2σ(*F*^2^)] = 0.044	H-atom parameters constrained
*wR*(*F*^2^) = 0.106	*w* = 1/[σ^2^(*F*_o_^2^) + (0.0438*P*)^2^ + 1.5689*P*] where *P* = (*F*_o_^2^ + 2*F*_c_^2^)/3
*S* = 1.05	(Δ/σ)_max_ = 0.001
6571 reflections	Δρ_max_ = 0.47 e Å^−^^3^
581 parameters	Δρ_min_ = −0.52 e Å^−^^3^

### Special details

**Table:**

Geometry. All esds (except the esd in the dihedral angle between two l.s. planes) are estimated using the full covariance matrix. The cell esds are taken into account individually in the estimation of esds in distances, angles and torsion angles; correlations between esds in cell parameters are only used when they are defined by crystal symmetry. An approximate (isotropic) treatment of cell esds is used for estimating esds involving l.s. planes.

### Fractional atomic coordinates and isotropic or equivalent isotropic displacement parameters (Å^2^)

**Table:**

	*x*	*y*	*z*	*U*_iso_*/*U*_eq_	
S1	0.40491 (7)	0.27322 (7)	0.33986 (2)	0.02327 (15)	
S2	1.17808 (7)	0.29094 (7)	0.15763 (2)	0.02404 (15)	
F1	0.6784 (2)	−0.21313 (19)	0.28932 (6)	0.0473 (4)	
F2	0.6798 (2)	−0.2261 (2)	0.36604 (6)	0.0526 (5)	
F3	0.0164 (2)	0.53003 (19)	0.34514 (6)	0.0477 (4)	
F4	0.18482 (19)	0.53143 (16)	0.28142 (5)	0.0326 (3)	
F5	−0.0713 (2)	0.51888 (19)	0.27738 (6)	0.0465 (4)	
F6	0.6485 (2)	0.5512 (2)	0.21727 (6)	0.0501 (4)	
F7	0.6787 (3)	0.5868 (2)	0.13991 (7)	0.0629 (5)	
F8	1.4342 (2)	−0.0947 (2)	0.15363 (6)	0.0505 (5)	
F9	1.39010 (18)	0.03048 (19)	0.21686 (5)	0.0381 (4)	
F10	1.3647 (2)	−0.22070 (19)	0.22130 (6)	0.0523 (5)	
O1	0.4016 (2)	−0.06137 (18)	0.33033 (6)	0.0292 (4)	
O2	0.3455 (2)	0.42426 (19)	0.35764 (6)	0.0343 (4)	
O3	0.5333 (2)	0.2632 (2)	0.30221 (6)	0.0356 (4)	
O4	0.3555 (3)	−0.3031 (3)	0.52168 (8)	0.0591 (6)	
O5	0.0298 (3)	0.3079 (3)	0.51945 (7)	0.0460 (5)	
O6	0.8254 (2)	0.2966 (2)	0.17644 (6)	0.0309 (4)	
O7	1.1568 (2)	0.3944 (2)	0.19444 (6)	0.0349 (4)	
O8	1.3393 (2)	0.2352 (2)	0.13777 (6)	0.0334 (4)	
O9	0.7015 (3)	0.3776 (3)	−0.02223 (8)	0.0535 (6)	
O10	1.3171 (3)	0.0395 (3)	−0.02043 (7)	0.0449 (5)	
N1	0.4795 (2)	0.1502 (2)	0.38497 (7)	0.0250 (4)	
H1N	0.5842	0.1083	0.3822	0.030*	
N2	0.4113 (3)	−0.0440 (2)	0.44874 (8)	0.0315 (5)	
N3	0.2718 (3)	0.2139 (2)	0.44832 (7)	0.0293 (5)	
N4	0.2201 (3)	0.1158 (3)	0.48799 (7)	0.0360 (5)	
N5	0.0657 (4)	0.0425 (4)	0.55905 (8)	0.0537 (7)	
N6	1.0775 (3)	0.3946 (2)	0.11402 (7)	0.0273 (5)	
H6N	1.0315	0.4945	0.1175	0.033*	
N7	0.9191 (3)	0.3767 (2)	0.05042 (7)	0.0313 (5)	
N8	1.1790 (3)	0.2324 (3)	0.05044 (7)	0.0303 (5)	
N9	1.1034 (3)	0.2116 (3)	0.01107 (7)	0.0354 (5)	
N10	1.0719 (3)	0.1088 (3)	−0.05969 (8)	0.0483 (6)	
C1	0.4275 (3)	−0.2344 (3)	0.33482 (10)	0.0319 (6)	
H1A	0.3768	−0.2749	0.3091	0.038*	
H1B	0.3776	−0.2797	0.3661	0.038*	
C2	0.6090 (4)	−0.2821 (3)	0.33091 (10)	0.0366 (7)	
H2	0.6349	−0.4030	0.3329	0.044*	
C3	0.2487 (3)	0.0195 (3)	0.32428 (8)	0.0226 (5)	
C4	0.1131 (3)	−0.0560 (3)	0.31745 (9)	0.0283 (6)	
H4	0.1223	−0.1710	0.3198	0.034*	
C5	−0.0334 (3)	0.0372 (3)	0.30731 (9)	0.0319 (6)	
H5	−0.1263	−0.0142	0.3032	0.038*	
C6	−0.0480 (3)	0.2044 (3)	0.30301 (9)	0.0291 (6)	
H6	−0.1488	0.2671	0.2944	0.035*	
C7	0.0837 (3)	0.2816 (3)	0.31124 (8)	0.0219 (5)	
C8	0.2322 (3)	0.1891 (3)	0.32403 (7)	0.0188 (5)	
C9	0.0556 (3)	0.4652 (3)	0.30368 (9)	0.0303 (6)	
C10	0.3842 (3)	0.1093 (3)	0.42737 (8)	0.0264 (5)	
C11	0.3046 (3)	−0.0355 (3)	0.48755 (9)	0.0303 (6)	
C12	0.2670 (4)	−0.1556 (4)	0.52547 (11)	0.0451 (8)	
C13	0.1508 (4)	−0.1123 (4)	0.55917 (10)	0.0472 (8)	
H13	0.1246	−0.1919	0.5848	0.057*	
C14	0.1007 (4)	0.1546 (4)	0.52415 (9)	0.0400 (7)	
C15	0.3152 (5)	−0.4323 (4)	0.55754 (13)	0.0761 (13)	
H15A	0.3362	−0.4064	0.5890	0.114*	
H15B	0.3837	−0.5338	0.5506	0.114*	
H15C	0.1981	−0.4448	0.5575	0.114*	
C16	−0.0932 (4)	0.3529 (4)	0.55773 (11)	0.0618 (10)	
H16A	−0.1876	0.2927	0.5580	0.093*	
H16B	−0.1309	0.4697	0.5526	0.093*	
H16C	−0.0443	0.3266	0.5884	0.093*	
C17	0.6501 (3)	0.3241 (3)	0.17727 (11)	0.0388 (7)	
H17A	0.5996	0.2640	0.2062	0.047*	
H17B	0.6132	0.2872	0.1487	0.047*	
C18	0.6008 (4)	0.5018 (4)	0.17763 (12)	0.0460 (8)	
H18	0.4782	0.5284	0.1767	0.055*	
C19	0.8998 (3)	0.1400 (3)	0.17952 (8)	0.0244 (5)	
C20	0.8126 (3)	0.0072 (3)	0.18630 (9)	0.0326 (6)	
H20	0.6953	0.0231	0.1871	0.039*	
C21	0.8963 (3)	−0.1464 (3)	0.19184 (9)	0.0350 (6)	
H21	0.8369	−0.2372	0.1948	0.042*	
C22	1.0651 (3)	−0.1711 (3)	0.19321 (9)	0.0321 (6)	
H22	1.1208	−0.2783	0.1991	0.039*	
C23	1.1547 (3)	−0.0404 (3)	0.18604 (8)	0.0238 (5)	
C24	1.0729 (3)	0.1177 (3)	0.17622 (8)	0.0203 (5)	
C25	1.3353 (3)	−0.0779 (3)	0.19369 (9)	0.0344 (6)	
C26	1.0613 (3)	0.3314 (3)	0.07141 (8)	0.0257 (5)	
C27	0.9510 (3)	0.2979 (3)	0.01135 (8)	0.0275 (6)	
C28	0.8526 (4)	0.2892 (3)	−0.02617 (10)	0.0407 (7)	
C29	0.9154 (4)	0.1956 (4)	−0.05974 (9)	0.0422 (7)	
H29	0.8498	0.1880	−0.0850	0.051*	
C30	1.1653 (4)	0.1154 (3)	−0.02503 (9)	0.0369 (7)	
C31	0.5908 (5)	0.3640 (5)	−0.05730 (12)	0.0651 (10)	
H31A	0.6383	0.4036	−0.0891	0.098*	
H31B	0.4841	0.4291	−0.0501	0.098*	
H31C	0.5747	0.2500	−0.0567	0.098*	
C32	1.3861 (4)	−0.0568 (4)	−0.05853 (11)	0.0574 (9)	
H32A	1.3238	−0.1487	−0.0580	0.086*	
H32B	1.5023	−0.0976	−0.0540	0.086*	
H32C	1.3789	0.0106	−0.0894	0.086*	

### Atomic displacement parameters (Å^2^)

**Table:**

	*U*^11^	*U*^22^	*U*^33^	*U*^12^	*U*^13^	*U*^23^
S1	0.0248 (3)	0.0191 (3)	0.0273 (3)	−0.0096 (2)	−0.0074 (2)	0.0035 (2)
S2	0.0227 (3)	0.0273 (3)	0.0243 (3)	−0.0104 (3)	−0.0056 (2)	−0.0001 (2)
F1	0.0495 (11)	0.0363 (9)	0.0517 (10)	−0.0063 (8)	0.0157 (8)	−0.0038 (8)
F2	0.0434 (11)	0.0549 (11)	0.0572 (11)	0.0058 (8)	−0.0152 (9)	−0.0032 (9)
F3	0.0608 (12)	0.0387 (9)	0.0427 (9)	0.0094 (8)	−0.0013 (8)	−0.0212 (7)
F4	0.0448 (9)	0.0202 (7)	0.0334 (8)	−0.0065 (6)	−0.0109 (7)	0.0034 (6)
F5	0.0383 (10)	0.0389 (9)	0.0598 (11)	0.0152 (7)	−0.0215 (8)	−0.0052 (8)
F6	0.0374 (10)	0.0509 (10)	0.0675 (12)	−0.0021 (8)	−0.0103 (8)	−0.0271 (9)
F7	0.0708 (14)	0.0459 (10)	0.0689 (13)	0.0095 (10)	−0.0230 (11)	0.0004 (9)
F8	0.0353 (10)	0.0617 (11)	0.0460 (10)	0.0086 (8)	0.0167 (8)	−0.0063 (8)
F9	0.0239 (8)	0.0547 (10)	0.0349 (8)	−0.0035 (7)	−0.0082 (6)	0.0007 (7)
F10	0.0421 (11)	0.0414 (9)	0.0616 (11)	0.0160 (8)	−0.0023 (8)	0.0140 (8)
O1	0.0291 (10)	0.0136 (8)	0.0455 (10)	−0.0016 (7)	−0.0089 (8)	−0.0020 (7)
O2	0.0486 (12)	0.0161 (8)	0.0417 (10)	−0.0081 (8)	−0.0193 (9)	−0.0008 (7)
O3	0.0253 (10)	0.0465 (11)	0.0347 (10)	−0.0149 (8)	−0.0016 (8)	0.0073 (8)
O4	0.0559 (15)	0.0501 (14)	0.0696 (15)	−0.0188 (12)	−0.0251 (12)	0.0290 (11)
O5	0.0544 (14)	0.0528 (13)	0.0349 (11)	−0.0212 (11)	0.0097 (9)	−0.0158 (9)
O6	0.0160 (9)	0.0283 (9)	0.0490 (11)	−0.0019 (7)	−0.0057 (8)	−0.0056 (8)
O7	0.0458 (12)	0.0342 (10)	0.0298 (10)	−0.0159 (9)	−0.0095 (8)	−0.0062 (8)
O8	0.0191 (9)	0.0468 (11)	0.0346 (10)	−0.0121 (8)	−0.0033 (7)	0.0041 (8)
O9	0.0629 (16)	0.0460 (12)	0.0560 (13)	−0.0108 (11)	−0.0342 (11)	0.0043 (10)
O10	0.0409 (13)	0.0634 (13)	0.0343 (11)	−0.0178 (11)	0.0065 (9)	−0.0158 (10)
N1	0.0177 (11)	0.0273 (11)	0.0302 (11)	−0.0045 (8)	−0.0094 (8)	0.0041 (9)
N2	0.0320 (13)	0.0284 (11)	0.0357 (12)	−0.0117 (10)	−0.0146 (10)	0.0086 (9)
N3	0.0346 (13)	0.0310 (11)	0.0249 (11)	−0.0144 (10)	−0.0046 (9)	−0.0004 (9)
N4	0.0387 (14)	0.0455 (14)	0.0273 (12)	−0.0188 (11)	−0.0086 (10)	0.0014 (10)
N5	0.0623 (19)	0.079 (2)	0.0285 (13)	−0.0423 (16)	−0.0065 (12)	0.0003 (13)
N6	0.0355 (13)	0.0201 (10)	0.0276 (11)	−0.0067 (9)	−0.0086 (9)	0.0003 (8)
N7	0.0346 (13)	0.0272 (11)	0.0332 (12)	−0.0091 (10)	−0.0120 (10)	0.0055 (9)
N8	0.0332 (13)	0.0358 (12)	0.0242 (11)	−0.0126 (10)	−0.0043 (9)	−0.0018 (9)
N9	0.0446 (15)	0.0384 (13)	0.0262 (12)	−0.0190 (11)	−0.0053 (10)	0.0026 (10)
N10	0.0610 (15)	0.0614 (17)	0.0280 (12)	−0.0318 (12)	−0.0021 (12)	−0.0012 (11)
C1	0.0404 (16)	0.0120 (11)	0.0417 (15)	−0.0024 (11)	−0.0001 (12)	−0.0003 (10)
C2	0.0409 (17)	0.0201 (13)	0.0451 (17)	0.0000 (12)	0.0027 (13)	0.0004 (12)
C3	0.0243 (14)	0.0218 (12)	0.0212 (12)	−0.0040 (10)	−0.0013 (10)	0.0005 (9)
C4	0.0325 (15)	0.0232 (12)	0.0317 (14)	−0.0128 (11)	−0.0013 (11)	−0.0039 (10)
C5	0.0253 (14)	0.0408 (15)	0.0337 (14)	−0.0175 (12)	0.0000 (11)	−0.0069 (12)
C6	0.0192 (13)	0.0387 (15)	0.0300 (13)	−0.0029 (11)	−0.0016 (10)	−0.0075 (11)
C7	0.0218 (13)	0.0239 (12)	0.0199 (11)	−0.0014 (10)	−0.0008 (9)	−0.0049 (9)
C8	0.0214 (13)	0.0177 (11)	0.0180 (11)	−0.0064 (9)	−0.0005 (9)	−0.0011 (9)
C9	0.0319 (15)	0.0264 (13)	0.0319 (14)	0.0070 (11)	−0.0075 (11)	−0.0090 (11)
C10	0.0281 (14)	0.0276 (13)	0.0268 (13)	−0.0126 (11)	−0.0119 (11)	0.0021 (10)
C11	0.0300 (15)	0.0359 (15)	0.0285 (14)	−0.0172 (12)	−0.0143 (11)	0.0068 (11)
C12	0.0468 (19)	0.0523 (19)	0.0403 (17)	−0.0251 (16)	−0.0251 (15)	0.0175 (14)
C13	0.056 (2)	0.065 (2)	0.0266 (15)	−0.0373 (18)	−0.0174 (14)	0.0175 (14)
C14	0.0433 (18)	0.059 (2)	0.0240 (14)	−0.0256 (15)	−0.0038 (12)	−0.0066 (13)
C15	0.099 (3)	0.062 (2)	0.072 (2)	−0.043 (2)	−0.039 (2)	0.0379 (19)
C16	0.071 (2)	0.078 (2)	0.0443 (19)	−0.036 (2)	0.0250 (17)	−0.0330 (18)
C17	0.0167 (14)	0.0439 (16)	0.0591 (18)	−0.0001 (12)	−0.0096 (12)	−0.0178 (14)
C18	0.0244 (16)	0.0511 (18)	0.067 (2)	0.0050 (14)	−0.0176 (14)	−0.0227 (16)
C19	0.0220 (13)	0.0267 (13)	0.0252 (12)	−0.0033 (10)	−0.0033 (10)	−0.0045 (10)
C20	0.0242 (14)	0.0402 (15)	0.0362 (15)	−0.0136 (12)	−0.0006 (11)	−0.0065 (12)
C21	0.0431 (18)	0.0301 (14)	0.0355 (15)	−0.0191 (13)	0.0012 (12)	−0.0059 (11)
C22	0.0437 (17)	0.0238 (13)	0.0280 (13)	−0.0046 (12)	0.0018 (12)	−0.0036 (10)
C23	0.0251 (13)	0.0263 (13)	0.0185 (11)	−0.0025 (10)	0.0038 (10)	−0.0020 (10)
C24	0.0182 (12)	0.0238 (12)	0.0195 (11)	−0.0059 (10)	−0.0009 (9)	−0.0022 (9)
C25	0.0301 (15)	0.0367 (15)	0.0312 (14)	0.0040 (12)	0.0026 (12)	0.0019 (12)
C26	0.0300 (14)	0.0230 (12)	0.0254 (13)	−0.0114 (11)	−0.0061 (11)	0.0043 (10)
C27	0.0373 (16)	0.0241 (13)	0.0230 (13)	−0.0144 (11)	−0.0087 (11)	0.0072 (10)
C28	0.053 (2)	0.0354 (15)	0.0367 (16)	−0.0185 (14)	−0.0211 (14)	0.0123 (13)
C29	0.0613 (17)	0.0504 (18)	0.0212 (13)	−0.0282 (13)	−0.0155 (13)	0.0046 (12)
C30	0.0489 (19)	0.0424 (16)	0.0233 (14)	−0.0242 (14)	0.0030 (12)	−0.0027 (12)
C31	0.066 (3)	0.073 (2)	0.062 (2)	−0.022 (2)	−0.0397 (19)	0.0113 (18)
C32	0.056 (2)	0.081 (2)	0.0413 (18)	−0.0272 (19)	0.0199 (16)	−0.0260 (17)

### Geometric parameters (Å, º)

**Table:**

S1—O3	1.4217 (18)	N10—C30	1.316 (4)
S1—O2	1.4238 (18)	N10—C29	1.381 (4)
S1—N1	1.6466 (19)	C1—C2	1.481 (4)
S1—C8	1.787 (2)	C1—H1A	0.9900
S2—O7	1.4207 (18)	C1—H1B	0.9900
S2—O8	1.4210 (18)	C2—H2	1.0000
S2—N6	1.640 (2)	C3—C4	1.395 (3)
S2—C24	1.788 (2)	C3—C8	1.406 (3)
F1—C2	1.353 (3)	C4—C5	1.371 (4)
F2—C2	1.364 (3)	C4—H4	0.9500
F3—C9	1.346 (3)	C5—C6	1.379 (4)
F4—C9	1.330 (3)	C5—H5	0.9500
F5—C9	1.333 (3)	C6—C7	1.388 (3)
F6—C18	1.355 (3)	C6—H6	0.9500
F7—C18	1.361 (4)	C7—C8	1.401 (3)
F8—C25	1.336 (3)	C7—C9	1.513 (3)
F9—C25	1.335 (3)	C11—C12	1.417 (4)
F10—C25	1.348 (3)	C12—C13	1.324 (5)
O1—C3	1.353 (3)	C13—H13	0.9500
O1—C1	1.425 (3)	C15—H15A	0.9800
O4—C12	1.351 (4)	C15—H15B	0.9800
O4—C15	1.438 (4)	C15—H15C	0.9800
O5—C14	1.328 (4)	C16—H16A	0.9800
O5—C16	1.452 (3)	C16—H16B	0.9800
O6—C19	1.361 (3)	C16—H16C	0.9800
O6—C17	1.420 (3)	C17—C18	1.487 (4)
O9—C28	1.351 (4)	C17—H17A	0.9900
O9—C31	1.436 (4)	C17—H17B	0.9900
O10—C30	1.324 (4)	C18—H18	1.0000
O10—C32	1.450 (3)	C19—C20	1.389 (3)
N1—C10	1.397 (3)	C19—C24	1.399 (3)
N1—H1N	0.8800	C20—C21	1.367 (4)
N2—C11	1.336 (3)	C20—H20	0.9500
N2—C10	1.346 (3)	C21—C22	1.373 (4)
N3—C10	1.326 (3)	C21—H21	0.9500
N3—N4	1.373 (3)	C22—C23	1.386 (3)
N4—C11	1.355 (3)	C22—H22	0.9500
N4—C14	1.380 (4)	C23—C24	1.403 (3)
N5—C14	1.313 (4)	C23—C25	1.501 (4)
N5—C13	1.382 (4)	C27—C28	1.413 (4)
N6—C26	1.403 (3)	C28—C29	1.326 (4)
N6—H6N	0.8800	C29—H29	0.9500
N7—C27	1.344 (3)	C31—H31A	0.9800
N7—C26	1.346 (3)	C31—H31B	0.9800
N8—C26	1.323 (3)	C31—H31C	0.9800
N8—N9	1.370 (3)	C32—H32A	0.9800
N9—C27	1.350 (3)	C32—H32B	0.9800
N9—C30	1.393 (3)	C32—H32C	0.9800
			
O3—S1—O2	120.60 (11)	C12—C13—N5	123.4 (3)
O3—S1—N1	104.84 (11)	C12—C13—H13	118.3
O2—S1—N1	106.54 (10)	N5—C13—H13	118.3
O3—S1—C8	109.19 (10)	N5—C14—O5	125.9 (3)
O2—S1—C8	108.35 (11)	N5—C14—N4	119.6 (3)
N1—S1—C8	106.43 (10)	O5—C14—N4	114.5 (2)
O7—S2—O8	120.38 (11)	O4—C15—H15A	109.5
O7—S2—N6	104.97 (11)	O4—C15—H15B	109.5
O8—S2—N6	107.16 (11)	H15A—C15—H15B	109.5
O7—S2—C24	109.88 (10)	O4—C15—H15C	109.5
O8—S2—C24	107.92 (11)	H15A—C15—H15C	109.5
N6—S2—C24	105.54 (10)	H15B—C15—H15C	109.5
C3—O1—C1	120.10 (19)	O5—C16—H16A	109.5
C12—O4—C15	116.5 (3)	O5—C16—H16B	109.5
C14—O5—C16	115.5 (2)	H16A—C16—H16B	109.5
C19—O6—C17	117.77 (19)	O5—C16—H16C	109.5
C28—O9—C31	116.7 (3)	H16A—C16—H16C	109.5
C30—O10—C32	115.5 (2)	H16B—C16—H16C	109.5
C10—N1—S1	123.11 (17)	O6—C17—C18	106.7 (2)
C10—N1—H1N	118.4	O6—C17—H17A	110.4
S1—N1—H1N	118.4	C18—C17—H17A	110.4
C11—N2—C10	100.9 (2)	O6—C17—H17B	110.4
C10—N3—N4	100.3 (2)	C18—C17—H17B	110.4
C11—N4—N3	109.7 (2)	H17A—C17—H17B	108.6
C11—N4—C14	121.8 (2)	F6—C18—F7	105.9 (2)
N3—N4—C14	128.4 (2)	F6—C18—C17	110.2 (3)
C14—N5—C13	119.3 (3)	F7—C18—C17	111.1 (2)
C26—N6—S2	123.03 (17)	F6—C18—H18	109.9
C26—N6—H6N	118.5	F7—C18—H18	109.9
S2—N6—H6N	118.5	C17—C18—H18	109.9
C27—N7—C26	100.8 (2)	O6—C19—C20	123.2 (2)
C26—N8—N9	100.1 (2)	O6—C19—C24	116.2 (2)
C27—N9—N8	110.4 (2)	C20—C19—C24	120.5 (2)
C27—N9—C30	121.9 (2)	C21—C20—C19	119.7 (2)
N8—N9—C30	127.7 (2)	C21—C20—H20	120.1
C30—N10—C29	120.1 (3)	C19—C20—H20	120.1
O1—C1—C2	106.3 (2)	C20—C21—C22	120.8 (2)
O1—C1—H1A	110.5	C20—C21—H21	119.6
C2—C1—H1A	110.5	C22—C21—H21	119.6
O1—C1—H1B	110.5	C21—C22—C23	120.3 (2)
C2—C1—H1B	110.5	C21—C22—H22	119.8
H1A—C1—H1B	108.7	C23—C22—H22	119.8
F1—C2—F2	105.6 (2)	C22—C23—C24	119.8 (2)
F1—C2—C1	110.6 (2)	C22—C23—C25	116.3 (2)
F2—C2—C1	110.8 (2)	C24—C23—C25	123.7 (2)
F1—C2—H2	109.9	C19—C24—C23	118.3 (2)
F2—C2—H2	109.9	C19—C24—S2	118.21 (17)
C1—C2—H2	109.9	C23—C24—S2	123.49 (18)
O1—C3—C4	123.6 (2)	F9—C25—F8	108.1 (2)
O1—C3—C8	116.1 (2)	F9—C25—F10	105.0 (2)
C4—C3—C8	120.3 (2)	F8—C25—F10	105.2 (2)
C5—C4—C3	119.5 (2)	F9—C25—C23	113.5 (2)
C5—C4—H4	120.3	F8—C25—C23	114.2 (2)
C3—C4—H4	120.3	F10—C25—C23	110.2 (2)
C4—C5—C6	121.0 (2)	N8—C26—N7	118.5 (2)
C4—C5—H5	119.5	N8—C26—N6	123.6 (2)
C6—C5—H5	119.5	N7—C26—N6	117.9 (2)
C5—C6—C7	120.4 (2)	N7—C27—N9	110.3 (2)
C5—C6—H6	119.8	N7—C27—C28	131.1 (3)
C7—C6—H6	119.8	N9—C27—C28	118.6 (2)
C6—C7—C8	119.8 (2)	C29—C28—O9	128.1 (3)
C6—C7—C9	115.7 (2)	C29—C28—C27	117.8 (3)
C8—C7—C9	124.5 (2)	O9—C28—C27	114.0 (3)
C7—C8—C3	118.7 (2)	C28—C29—N10	122.9 (3)
C7—C8—S1	124.01 (17)	C28—C29—H29	118.5
C3—C8—S1	117.28 (18)	N10—C29—H29	118.5
F4—C9—F5	106.3 (2)	N10—C30—O10	126.7 (3)
F4—C9—F3	107.5 (2)	N10—C30—N9	118.7 (3)
F5—C9—F3	105.6 (2)	O10—C30—N9	114.6 (2)
F4—C9—C7	113.7 (2)	O9—C31—H31A	109.5
F5—C9—C7	111.2 (2)	O9—C31—H31B	109.5
F3—C9—C7	112.0 (2)	H31A—C31—H31B	109.5
N3—C10—N2	118.3 (2)	O9—C31—H31C	109.5
N3—C10—N1	123.7 (2)	H31A—C31—H31C	109.5
N2—C10—N1	118.0 (2)	H31B—C31—H31C	109.5
N2—C11—N4	110.8 (2)	O10—C32—H32A	109.5
N2—C11—C12	130.9 (3)	O10—C32—H32B	109.5
N4—C11—C12	118.2 (3)	H32A—C32—H32B	109.5
C13—C12—O4	128.3 (3)	O10—C32—H32C	109.5
C13—C12—C11	117.6 (3)	H32A—C32—H32C	109.5
O4—C12—C11	114.1 (3)	H32B—C32—H32C	109.5
			
O3—S1—N1—C10	−171.86 (18)	C16—O5—C14—N4	−178.4 (2)
O2—S1—N1—C10	59.2 (2)	C11—N4—C14—N5	−0.7 (4)
C8—S1—N1—C10	−56.2 (2)	N3—N4—C14—N5	178.7 (2)
C10—N3—N4—C11	0.3 (2)	C11—N4—C14—O5	179.8 (2)
C10—N3—N4—C14	−179.1 (2)	N3—N4—C14—O5	−0.9 (4)
O7—S2—N6—C26	−176.81 (19)	C19—O6—C17—C18	175.3 (2)
O8—S2—N6—C26	54.1 (2)	O6—C17—C18—F6	−61.3 (3)
C24—S2—N6—C26	−60.7 (2)	O6—C17—C18—F7	55.7 (3)
C26—N8—N9—C27	0.3 (2)	C17—O6—C19—C20	−4.0 (3)
C26—N8—N9—C30	−178.6 (2)	C17—O6—C19—C24	177.0 (2)
C3—O1—C1—C2	168.7 (2)	O6—C19—C20—C21	−176.2 (2)
O1—C1—C2—F1	−56.8 (3)	C24—C19—C20—C21	2.8 (4)
O1—C1—C2—F2	59.8 (3)	C19—C20—C21—C22	3.3 (4)
C1—O1—C3—C4	−6.4 (3)	C20—C21—C22—C23	−4.0 (4)
C1—O1—C3—C8	175.1 (2)	C21—C22—C23—C24	−1.5 (4)
O1—C3—C4—C5	−174.1 (2)	C21—C22—C23—C25	173.3 (2)
C8—C3—C4—C5	4.3 (3)	O6—C19—C24—C23	171.0 (2)
C3—C4—C5—C6	1.0 (4)	C20—C19—C24—C23	−8.1 (3)
C4—C5—C6—C7	−3.2 (4)	O6—C19—C24—S2	−11.3 (3)
C5—C6—C7—C8	0.0 (3)	C20—C19—C24—S2	169.70 (19)
C5—C6—C7—C9	177.8 (2)	C22—C23—C24—C19	7.4 (3)
C6—C7—C8—C3	5.3 (3)	C25—C23—C24—C19	−167.0 (2)
C9—C7—C8—C3	−172.4 (2)	C22—C23—C24—S2	−170.27 (18)
C6—C7—C8—S1	−175.15 (17)	C25—C23—C24—S2	15.3 (3)
C9—C7—C8—S1	7.2 (3)	O7—S2—C24—C19	71.2 (2)
O1—C3—C8—C7	171.1 (2)	O8—S2—C24—C19	−155.77 (18)
C4—C3—C8—C7	−7.5 (3)	N6—S2—C24—C19	−41.5 (2)
O1—C3—C8—S1	−8.5 (3)	O7—S2—C24—C23	−111.1 (2)
C4—C3—C8—S1	172.95 (18)	O8—S2—C24—C23	21.9 (2)
O3—S1—C8—C7	−107.6 (2)	N6—S2—C24—C23	136.22 (19)
O2—S1—C8—C7	25.5 (2)	C22—C23—C25—F9	−138.3 (2)
N1—S1—C8—C7	139.78 (19)	C24—C23—C25—F9	36.3 (3)
O3—S1—C8—C3	72.01 (19)	C22—C23—C25—F8	97.3 (3)
O2—S1—C8—C3	−154.89 (17)	C24—C23—C25—F8	−88.1 (3)
N1—S1—C8—C3	−40.7 (2)	C22—C23—C25—F10	−20.8 (3)
C6—C7—C9—F4	−138.2 (2)	C24—C23—C25—F10	153.8 (2)
C8—C7—C9—F4	39.5 (3)	N9—N8—C26—N7	0.1 (3)
C6—C7—C9—F5	−18.2 (3)	N9—N8—C26—N6	−178.7 (2)
C8—C7—C9—F5	159.5 (2)	C27—N7—C26—N8	−0.5 (3)
C6—C7—C9—F3	99.7 (3)	C27—N7—C26—N6	178.4 (2)
C8—C7—C9—F3	−82.6 (3)	S2—N6—C26—N8	−36.0 (3)
N4—N3—C10—N2	0.0 (3)	S2—N6—C26—N7	145.19 (18)
N4—N3—C10—N1	−178.3 (2)	C26—N7—C27—N9	0.6 (2)
C11—N2—C10—N3	−0.2 (3)	C26—N7—C27—C28	178.6 (2)
C11—N2—C10—N1	178.2 (2)	N8—N9—C27—N7	−0.7 (3)
S1—N1—C10—N3	−39.4 (3)	C30—N9—C27—N7	178.4 (2)
S1—N1—C10—N2	142.28 (19)	N8—N9—C27—C28	−178.9 (2)
C10—N2—C11—N4	0.4 (3)	C30—N9—C27—C28	0.2 (3)
C10—N2—C11—C12	178.9 (3)	C31—O9—C28—C29	3.1 (4)
N3—N4—C11—N2	−0.4 (3)	C31—O9—C28—C27	−175.4 (2)
C14—N4—C11—N2	179.0 (2)	N7—C27—C28—C29	−177.5 (2)
N3—N4—C11—C12	−179.2 (2)	N9—C27—C28—C29	0.2 (4)
C14—N4—C11—C12	0.3 (4)	N7—C27—C28—O9	1.2 (4)
C15—O4—C12—C13	2.7 (4)	N9—C27—C28—O9	178.9 (2)
C15—O4—C12—C11	−176.1 (2)	O9—C28—C29—N10	−179.1 (2)
N2—C11—C12—C13	−178.4 (3)	C27—C28—C29—N10	−0.7 (4)
N4—C11—C12—C13	0.1 (4)	C30—N10—C29—C28	0.7 (4)
N2—C11—C12—O4	0.6 (4)	C29—N10—C30—O10	−179.7 (2)
N4—C11—C12—O4	179.1 (2)	C29—N10—C30—N9	−0.3 (4)
O4—C12—C13—N5	−178.9 (3)	C32—O10—C30—N10	1.5 (4)
C11—C12—C13—N5	0.0 (4)	C32—O10—C30—N9	−178.0 (2)
C14—N5—C13—C12	−0.4 (4)	C27—N9—C30—N10	−0.2 (4)
C13—N5—C14—O5	−179.8 (3)	N8—N9—C30—N10	178.7 (2)
C13—N5—C14—N4	0.7 (4)	C27—N9—C30—O10	179.4 (2)
C16—O5—C14—N5	2.0 (4)	N8—N9—C30—O10	−1.8 (4)

### Hydrogen-bond geometry (Å, º)

**Table:**

*D*—H···*A*	*D*—H	H···*A*	*D*···*A*	*D*—H···*A*
C1—H1*A*···F10^i^	0.99	2.48	3.293 (3)	139
C16—H16*C*···F3^ii^	0.98	2.40	3.185 (3)	136
C17—H17*B*···O8^i^	0.99	2.40	3.102 (3)	127
C18—H18···F10^iii^	1.00	2.60	3.104 (3)	111
C20—H20···F9^i^	0.95	2.55	3.466 (3)	162
C32—H32*C*···F8^iv^	0.98	2.36	3.146 (3)	137

Symmetry codes: (i) *x*−1, *y*, *z*; (ii) −*x*, −*y*+1, −*z*+1; (iii) *x*−1, *y*+1, *z*; (iv) −*x*+3, −*y*, −*z*.
